# *PRNP *variation in UK sporadic and variant Creutzfeldt Jakob disease highlights genetic risk factors and a novel non-synonymous polymorphism

**DOI:** 10.1186/1471-2350-10-146

**Published:** 2009-12-26

**Authors:** Matthew T Bishop, Catherine Pennington, Craig A Heath, Robert G Will, Richard SG Knight

**Affiliations:** 1 National CJD Surveillance Unit, University of Edinburgh, Bryan Matthews Building, Western General Hospital, Crewe Road, Edinburgh, EH4 2XU, UK

## Abstract

**Background:**

Genetic analysis of the human prion protein gene (*PRNP*) in suspect cases of Creutzfeldt-Jakob disease (CJD) is necessary for accurate diagnosis and case classification. Previous publications on the genetic variation at the *PRNP *locus have highlighted the presence of numerous polymorphisms, in addition to the well recognised one at codon 129, with significant variability between geographically distinct populations. It is therefore of interest to consider their influence on susceptibility or the clinico-pathological disease phenotype. This study aimed to characterise the frequency and effect of *PRNP *open reading frame polymorphisms other than codon 129 in both disease and control samples sourced from the United Kingdom population.

**Methods:**

DNA was extracted from blood samples and genetic data obtained by full sequence analysis of the prion protein gene or by restriction fragment length polymorphism analysis using restriction enzymes specific to the gene polymorphism under investigation.

**Results:**

147 of 166 confirmed cases of variant CJD (vCJD) in the UK have had *PRNP *codon 129 genotyping and all are methionine homozygous at codon 129; 118 have had full *PRNP *gene sequencing. Of the latter, 5 cases have shown other polymorphic loci: at codon 219 (2, 1.69%), at codon 202 (2, 1.69%), and a 24 bp deletion in the octapeptide repeat region (1, 0.85%). E219K and D202D were not found in sporadic CJD (sCJD) cases and therefore may represent genetic risk factors for vCJD.

Genetic analysis of 309 confirmed UK sCJD patients showed codon 129 genotype frequencies of MM: 59.5% (n = 184), MV: 21.4% (n = 66), and VV: 19.1% (n = 59). Thirteen (4.2%) had the A117A polymorphism, one of which also had the P68P polymorphism, four (1.3%) had a 24 bp deletion, and a single patient had a novel missense variation at codon 167. As the phenotype of this latter case is similar to sCJD and in the absence of a family history of CJD, it is unknown whether this is a form of genetic CJD, or simply a neutral polymorphism.

**Conclusions:**

This analysis of *PRNP *genetic variation in UK CJD patients is the first to show a comprehensive comparison with healthy individuals (n = 970) from the same population, who were genotyped for the three most common variations (codon 129, codon 117, and 24 bp deletion). These latter two genetic variations were equally frequent in UK sCJD or vCJD cases and a normal (healthy blood donor) UK population.

## Background

At the time of this study, there were 166 confirmed cases of vCJD in the UK, and a further 44 patients in other countries. (For current UK and worldwide figures see http://www.cjd.ed.ac.uk) The widely accepted hypothesis is that vCJD was initially acquired through dietary infection with bovine produce contaminated with the infectious agent of bovine spongiform encephalopathy (BSE) [1,2]. Additionally, secondary cases have resulted from blood transfusion [3,4]. The proposed agent (designated a 'prion') has not been fully characterised but disease and infectivity are generally associated with an abnormally folded form (designated PrP^Sc^) of the host encoded prion protein (PrP^C^) [5]. Variant CJD shows marked differences in clinical presentation [6] and neuropathology [7] when compared with sporadic CJD (sCJD) which has a worldwide distribution affecting approximately 1-2 cases per million population per year and has an unknown aetiology. In comparison to sCJD, vCJD typically occurs in younger individuals (median ages of onset: 66 & 26 years respectively) and with a longer disease duration (median durations: 4 and 14 months respectively). There are two other forms of CJD: genetic and iatrogenic. Genetic CJD (gCJD) is an autosomal dominant inherited illness related to underlying mutations of the prion protein gene (*PRNP*). There are other genetic human prion diseases (Fatal Familial Insomnia (FFI), and Gerstmann Sträussler Scheinker syndrome (GSS)) traditionally separated from gCJD on clinico-pathological grounds, but which are related by certain common core features and an underlying causal *PRNP *mutation. Iatrogenic CJD (iCJD) results from the accidental person-to-person (medical or surgical) transmission of other forms of CJD.

Genetic analysis of the sCJD and vCJD disease cohort has an important role in accurate diagnostic classification, especially as genetic prion disease cannot always be differentiated clinically from other forms of prion disease and a family history is absent in up to about 50% of cases [8]. The gene coding for prion protein, *PRNP*, is a key target for analysis. This gene is highly conserved between mammalian species indicating an important biological role for the prion protein, but *PRNP *shows significant variation at the individual level in humans [9]. Over twenty missense and nonsense mutations have been identified in the open reading frame (ORF) that have been linked to genetic prion disease phenotypes [10]. Other polymorphic sites in *PRNP *(over twenty examples) that are not directly linked to a disease phenotype have been identified through analysis of suspected CJD cases and other populations. These polymorphisms may have disease modulating capacities and these effects may vary according to the population under investigation. The conversion of PrP^C ^to PrP^Sc ^is thought to involve some form of oligomerisation and the melting and refolding of the peptide chains. It is therefore possible that any changes to the amino-acid sequence could alter the thermodynamic stability of the protein and affect the kinetics of structural conversions [11].

The *PRNP *polymorphic residue at codon 129 (ATG-methionine to GTG-valine, M129V) has been studied extensively. Variation in the genotype frequencies occurs according to geographical region. In most countries studied, the MM and MV frequencies are approximately 40-50% however there is a large difference seen in Japan where the normal population frequencies are: MM 92%, MV 8%, and VV 0% [12]. Genotype data from other countries suggest a gradual increase in MM genotype frequency from West to East which may reflect the historical human migrations [12]. It has been shown that M129V may affect susceptibility to prion diseases [13], the incubation period in acquired forms [14,15] and the clinico-pathological phenotype [16]. Codon 129 homozygosity is considered a risk factor for human prion disease: while ~40% of the normal population is MM, ~70% of sCJD patients, and all clinical vCJD patients tested to date have this genotype [17]. Homozygosity has also been shown to be a risk factor, and reduces the incubation time, for both human growth hormone associated iCJD [14] and kuru [15] (an historical disease of the Fore linguistic tribe of Papua New Guinea transmitted by endocannibalism). In some circumstances, the genotype at codon 129 can determine the disease phenotype for human genetic prion cases associated with pathogenic mutations [16]. In addition, variation in sCJD clinico-pathological phenotype can be associated with specific genotypes [18].

The most significant finding with respect to codon 129 genotype and CJD is that all clinically probable and neuropathologically confirmed cases of vCJD so far analysed world-wide (n = 147 UK and 44 non-UK) have the MM genotype and, therefore, this genotype is categorised as a risk factor. However, there is evidence that other 129 genotypes are susceptible to BSE/vCJD infection and that they may develop disease after a longer incubation period than in MM individuals. It is hypothesised that MV and VV cases will occur in the future [19]. There are four reports of transmission of vCJD infection via blood transfusion from blood donors who later developed vCJD [3,4,20,21]. In three instances, the recipients developed clinical vCJD and all three were homozygous MM at codon 129. In the fourth case, there was no clinical or neuropathological evidence of vCJD in the recipient, but disease-related abnormal prion protein was found in the spleen and a lymph node. This individual was MV at codon 129 and may represent subclinical infection in a non-MM individual; it is impossible to know whether the individual would have developed clinical vCJD if they had lived longer [21]. In addition, two of three samples in an anonymous appendix study in the UK that were positive for deposition of prion disease associated PrP^Sc ^were genotyped as VV [22].

Variation in the DNA sequence of *PRNP *may be linked to other genetic changes separate to the prion protein itself, such as promoter elements or regions more distant to the *PRNP *gene. To understand more fully the control mechanisms of *PRNP *gene expression, such as promoter activity, extensive studies have been undertaken to examine the region of chromosome 20 adjacent to the *PRNP *locus [23-26]. Analysis of a 4.8 kb region around *PRNP*, identified 3 polymorphisms in areas that were predicted to control gene expression [24].

This study involved the genetic analysis of CJD cases referred to the National CJD Surveillance Unit during routine UK surveillance and a specific project to determine normal population *PRNP *polymorphisms in the United Kingdom.

## Methods

### Control DNA Samples

The Scottish Blood Donor controls (n = 778) were supplied as 0.5 ml aliquots of frozen whole blood by Dr Ian MacGregor (National Science Laboratory, Scottish National Blood Transfusion Service) from samples taken as part of a human prion disease study of blood markers (covered by Multi-Centre Research Ethics Committee for Scotland approval, reference MREC/02/10/46). There was no ethical approval for full *PRNP *sequence analysis of these samples.

DNA samples (UK DNA controls, n = 192) were purchased from the European Collection of Cell Cultures (ECACC) originating from donors in the UK cities of Oxford and Birmingham. (Catalogue references: HRC-1 and HRC-2.)

For the normal population data, the Scottish Blood Donor and UK DNA controls were genotyped for polymorphisms at M129V, and A117A, and the 24 bp deletion in the N-terminus octapeptide repeat region (the three most common polymorphic loci of *PRNP*). For the CJD cases, we had *PRNP *sequence analysis data on 118 vCJD and 309 sCJD patients.

### Patient Samples

Patient blood samples were taken from individuals under clinical review for suspected CJD, by venipuncture, into citrate anticoagulant treated blood collection tubes. Once received into the laboratory the blood was separated into its major constituents as follows:

1. Whole blood was spun at 450 g for 10 minutes to separate platelet rich plasma, buffy coat, and red blood cells (RBC).

2. Platelets were removed from the plasma by two washes in phosphate buffered saline (PBS) with centrifugations at 16,000 g leaving platelet poor plasma. This was stored at -80°C.

3. The buffy coat fraction was cleared of contaminating platelets by centrifugation at 180 g for 10 minutes, then the RBC were removed by lysis with distilled water and centrifugation at 180 g for 10 minutes. Finally the buffy coat fraction was washed in PBS then centrifuged at 100 g for 10 minutes before storing at -80°C.

4. The RBC were washed twice with PBS, with centrifugation at 180 g for 10 minutes each time. RBC were stored at -80°C as a 50% solution in PBS.

### DNA Extraction

DNA from cases and controls was prepared from 200 μl of frozen whole blood by cell lysis and column purification using the DNA Blood Mini Kit (Qiagen, UK) and stored at -20°C.

### PCR-RFLP Analysis

Amplification of the *PRNP *gene sequence (NCBI Accession: AL133396) by the polymerase chain reaction (PCR) involved forward primer (5'-TGA TAC CAT TGC TAT GCA CTC ATT C-3') and reverse primer (5'-GAC ACC ACC ACT AAA AGG GCT GCA G-3') at 5 pmoles each per reaction (Eurofins MWG Operon, Germany), that are specific for a 956 bp sequence. Each reaction contained 2 mM MgCl_2 _(Qiagen, UK), 0.2 mM dNTPs (Promega, UK), and 1 Unit of Taq Polymerase (HotStarTaq, Qiagen, UK). The thermal cycling program included an annealing temperature step-down from 65°C to 60°C over ten cycles followed by 30 cycles at 60°C.

**Figure 1:**
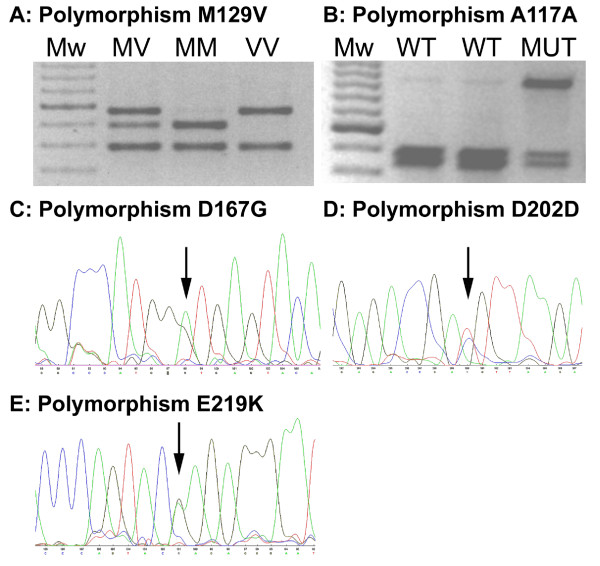
**Detection of *PRNP *polymorphisms**. Panels A and B: Genotyping by PCR amplification of *PRNP *and restriction enzyme digest. (Mw: 100 bp molecular weight ladder (dark band 600 bp); MV, MM, VV: codon 129 genotypes; WT: wild-type codon 117 genotype; MUT: heterozygous for codon 117 polymorphism) Panels C, D, and E: Electropherograms for polymorphisms detected by sequence analysis. (Arrows point to heterozygous base position.)

Confirmation of the codon 129 genotype was performed by restriction enzyme digestion at 37°C with NspI (New England Biolabs, UK). This enzyme cleaves the amplicon at *PRNP *codon 155 and at codon 129 only when the latter sequence codes for valine (-GTG-). This allowed for discrimination of the three genotypes: MM, MV, and VV by agarose gel electrophoresis and ethidium bromide staining, Figure 1. The presence of a 24 bp deletion in the octapeptide repeat region could be observed from the codon 129 genotyping agarose gel data due to an additional band shift for the restriction enzyme digest products. Codon 117 polymorphism genotype was determined by restriction enzyme digestion of the same *PRNP *PCR product with PvuII (New England Biolabs, UK) at 37°C. This enzyme cleaves the wild-type PCR product and not the codon 117 variant. In samples heterozygous for the codon 117 polymorphism this enzyme produces digest products of sizes 499 and 457 in addition to the full length 956 bp PCR amplicon. (Figure 1)

### Sequencing

If consent from the patient, or relative, had been obtained for full genetic analysis then the PCR product was sequenced by fluorescent dye-primer chemistry (Thermo Sequenase Primer Cycle Sequencing Kit, Amersham Biosciences, UK) using an ALF-Express DNA Sequencer (Amersham Biosciences, UK). PCR products were purified using a QIAquick PCR Purification Kit (Qiagen, UK) to remove excess primers and other PCR reagents. Four Cy5 labelled sequencing primers were used to give at least 2-fold coverage of the entire ORF (5'-AGG TGG CAC CCA CAG TCA GT-3'; 5'-CTA TGC ACT CAT TCA TTA TG-3'; 5'-CCT CAA GCT GGA AAA AGA TTA G-3'; 5'-CGA TAG TAA CGG TCC TCA TA-3'). Sequencing reaction products were electrophoresed through ReproGel Long Read acrylamide gels (Amersham Biosciences, UK). ALFwin Software (Amersham Biosciences, UK) was used to align the sequence data with a reference sequence and each codon of the ORF was visually checked by two individuals. This method allows for identification of novel polymorphisms rather than only checking for known ORF sequence changes. (Figure 1)

### Statistical Analysis

Analysis was undertaken through the use of R (v2.9.1) (R Development Core Team (2009). R: A language and environment for statistical computing. R Foundation for Statistical Computing, Vienna, Austria. ISBN 3-900051-07-0. URL: http://www.R-project.org).

## Results

### Variant CJD *PRNP *Sequence Data

Table 1 shows the results of *PRNP *gene sequence analysis in vCJD cases where material and specific consent was available. 147 confirmed cases were genotyped at codon 129 and all were methionine homozygotes (MM). Complete sequence analysis of the *PRNP *ORF was performed for 118 cases and found five individuals with genetic variation. Two had a synonymous (silent) polymorphism at codon 202 (GAC-aspartic acid to GAT-aspartic acid; D202D), two had a non-synonymous change at codon 219 (GAG-glutamic acid to AAG-lysine; E219K), and one had a 24 bp deletion (classified as DelR34 according to [27]). The DelR34 and the two D202D patients fulfilled the diagnostic criteria [28] for definite vCJD. The two patients with E219K genotype did not have a post mortem and were classified as probable vCJD. The E219K mutation has not been seen in white Caucasian populations and has so far only been detected in populations from Asian and the Pacific [29]. The two individuals with the E219K genotype found in this study were not of white Caucasian origin (the specific ancestral origin is unknown).

**Table 1:** *PRNP *gene sequence variation in vCJD cases

Codon	Number Tested	Genotype Data
129 (Met/Val)	147	All cases MM
	
	4 (blood transfusion associated infections)	MM (n = 3) MV (n = 1)*
	
	2 (appendix tissue)	VV (n = 2)**

202 (Asp/Asp)	118	DD (n = 2)

219 (Glu/Lys)	118	EK (n = 2)

24 bp deletion	118	DelR34 (n = 1)

* Non-clinical, non-neuropathologically confirmed case

** From anonymous screening program for vCJD associated PrP^Sc ^deposition

### Sporadic CJD Sequence Data

Sequence variation for 309 confirmed sCJD patients is shown in Table 2. Codon 129 genotyping produced allele frequencies similar to that expected for this disease from other studies [13,18] and to the overall figures published by the UK National CJD Surveillance Unit (MM 63%, MV 19%, VV 18%; n = 647) (http://www.cjd.ed.ac.uk; Annual Report 2008). Complete sequence analysis on these patients found thirteen with the silent polymorphism at codon 117 (GCA-alanine to GCG-alanine; A117A), which included one individual with an additional silent polymorphism at codon 68 (P68P), and four cases with a 24 bp deletion in the octapeptide repeat region. Of these 17 patients 13 were classified as definite and four as probable sCJD according to the diagnostic criteria. In addition, one patient was found to have a non-synonymous polymorphism at codon 167 (GAT-aspartic acid to GGT-glycine; D167G). There was nothing in the clinical phenotype suggesting that this could be a causative mutation of gCJD and the patient was classified as probable "sporadic CJD". Neuropathological analysis confirmed sporadic CJD with type 1 prion protein isoform present, and genetic analysis showed methionine homozygosity at codon 129 of *PRNP*.

**Table 2:** *PRNP *gene sequence variation in sCJD cases and controls

*PRNP *Variation	309 sCJD Patients	192 UK DNA Controls	778 Scottish Blood Donor Controls
Codon 129 (Met/Val)	MM (n = 184; 59.5%) MV (n = 66; 21.4%) VV (n = 59; 19.1%)	MM (n = 90; 46.9%) MV (n = 87; 45.3%) VV (n = 15; 7.8%)	MM (n = 337; 43.3%) MV (n = 344; 44.2%) VV (n = 97; 12.5%)

Codon 117 (Ala/Ala)	n = 13; 4.2% (10.4% of MV/VV cases)	n = 9; 4.7% (10.3% of MV/VV cases)	n = 47; 6.0% (10.7% of MV/VV cases)

24 bp Deletion	n = 4; 1.3% Deletion Class R34 (n = 3) R3 (n = 1)	n = 1; 0.5% Deletion Class R2 (n = 1)	n = 12; 1.5% Deletion Class R34 (n = 12)

Codon 167 (Asp/Gly)	DG (n = 1; 0.32%)	(no sequence data available)	(no sequence data available)

Codon 68 (Pro/Pro)	PP (n = 1; 0.32%)	(no sequence data available)	(no sequence data available)

### Control Codon 129 Genotype Data

**Figure 2:**
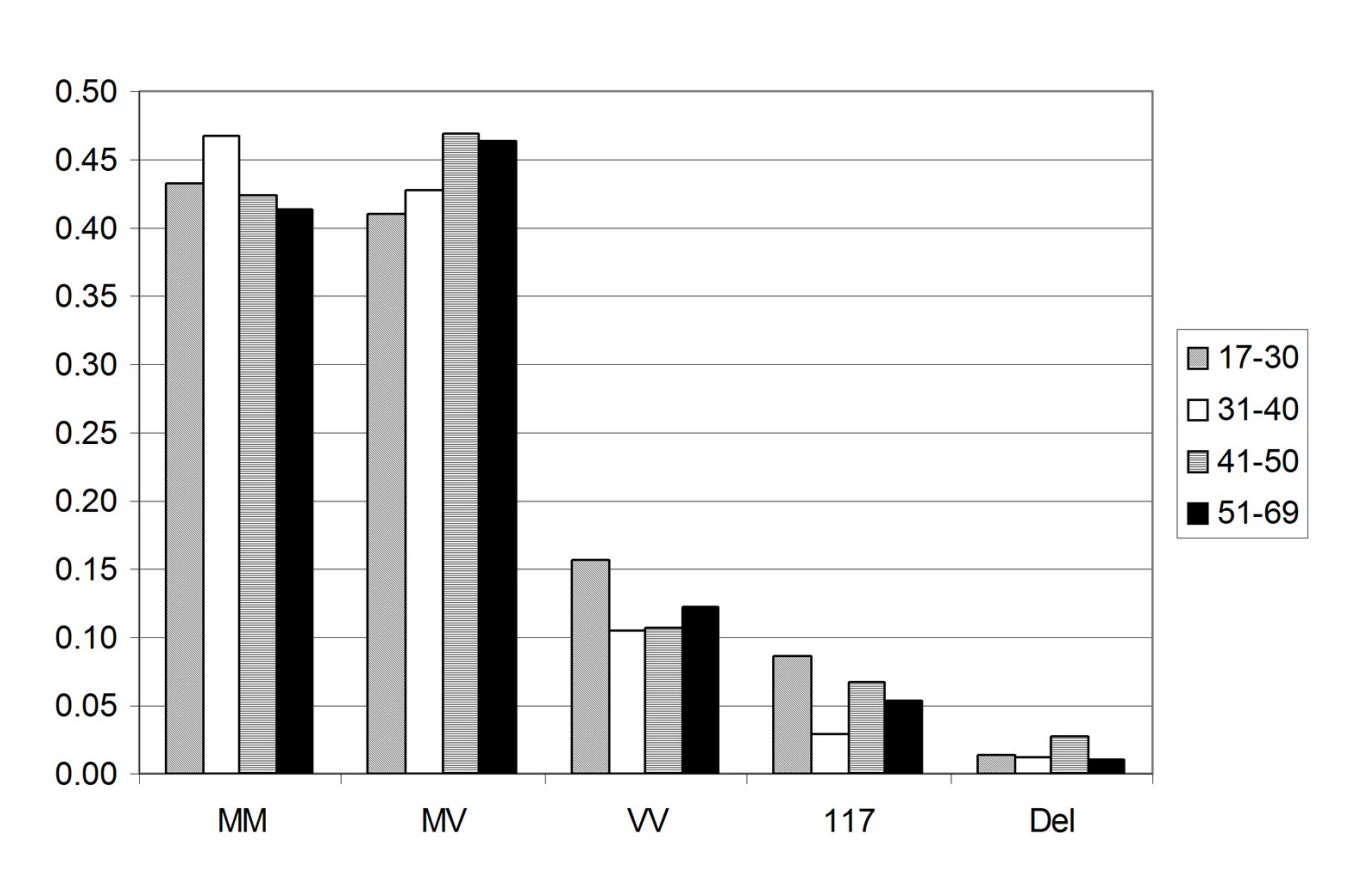
***PRNP *polymorphisms in Scottish Blood Donors**. Codon 129 and *PRNP *polymorphism frequency in relation to age of Scottish Blood Donors (n = 778).

**Figure 3:**
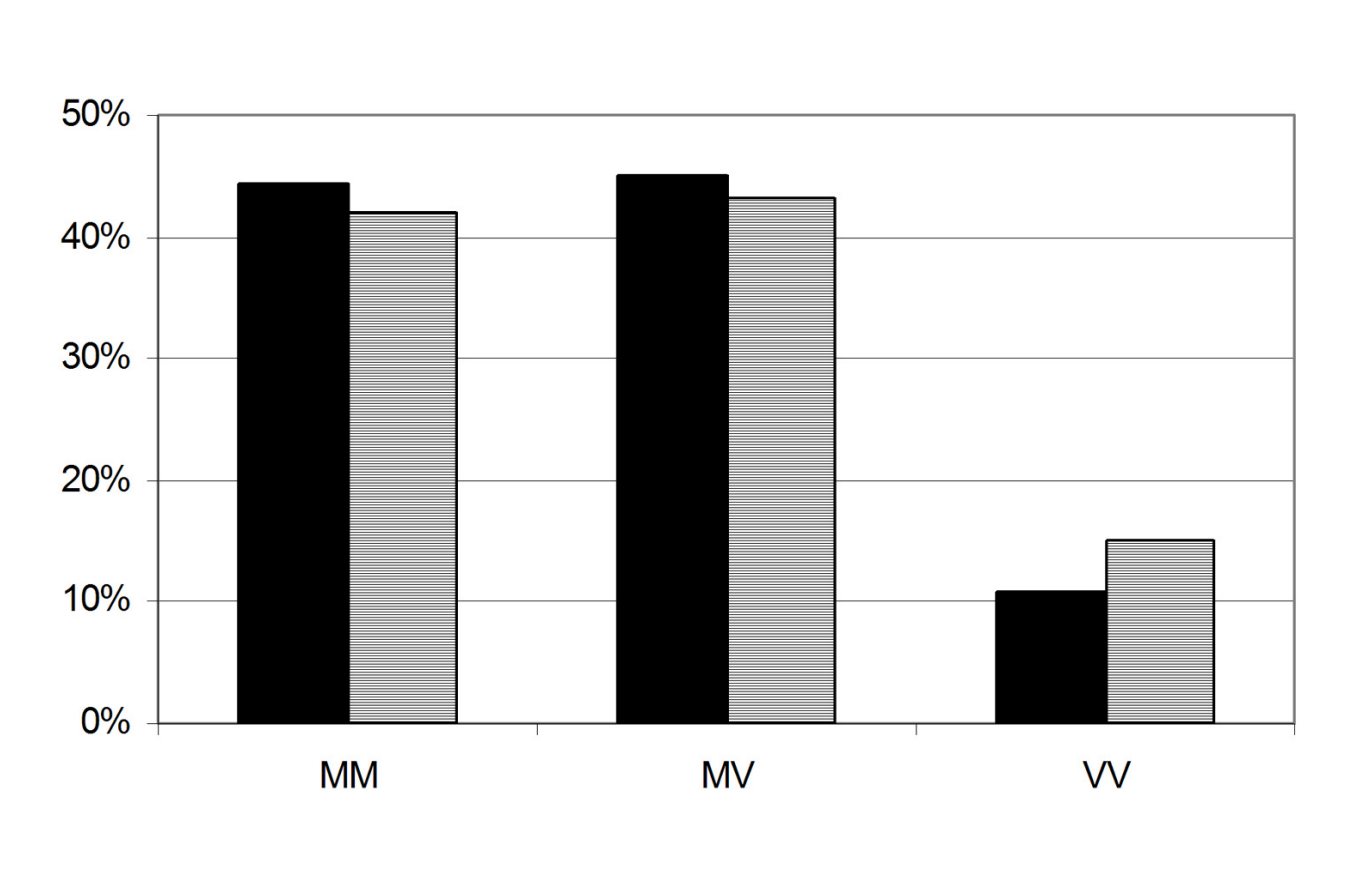
**Codon 129 and sex of Scottish Blood Donors**. Codon 129 genotype frequency in relation to sex of Scottish Blood Donors (n = 778, male = 456 (solid bars), female = 322 (hatched bars)).

Codon 129 genotype frequencies are given in Table 2 for samples from the UK DNA and Scottish Blood Donor controls. Together with the published Edinburgh/Belfast blood donors, the figures are similar across the three groups and combined (n = 1158) give the predicted UK national genotype frequencies of 44.1% (MM), 44.5% (MV), and 11.4% (VV). As there is a significant difference in age at onset between vCJD and sCJD, and that gender may influence sCJD survival [30], the Scottish Blood Donor data was stratified into four groups according to age (17-30, 31-40, 41-50, and 51-69), Figure 2, and by sex, Figure 3. Statistical analysis (Chi-Squared test) indicated that there were no age (p = 0.5774, 0.1270, and 0.4781) or gender (p = 0.2228, 0.9884, and 0.7527) effects determined by genotype at codon 129, or 117, or by the presence of a 24 bp deletion, respectively.

### Statistical Comparison (Chi Squared Testing) of Control and Case Data

Comparisons made between codon 129 genotype frequencies for sCJD, vCJD, and the two control groups showed statistically significant differences (P < 0.001, Table 3). There was no significant difference in genotype between the two control groups. There was no selection bias for the sequenced sCJD patients as there was no significant difference between that group and the larger group of UK National CJD Surveillance Unit sCJD patients that have been genotyped.

**Table 3:** Statistical analysis of *PRNP *polymorphism frequencies

	Chi-Squared Test - P value		
**Comparison**	**Codon 129**	**Codon 117**	**24 bp Deletion**

Sequenced sCJD (n = 309) vs. All NCJDSU sCJD cases (n = 647)	0.582	NA	NA

UK DNA Controls (n = 192) vs. Scottish Blood Donors (n = 778)	0.185	0.584	0.452*

Sequenced sCJD (n = 309) vs. UK DNA Controls (n = 192)	<0.001	0.975	0.700*

Sequenced sCJD (n = 309) vs. Scottish Blood Donors (n = 778)	<0.001	0.295	0.978*

Sequenced vCJD (n = 118) vs. Scottish Blood Donors (n = 778)	<0.001	NA	0.861*

*: Chi-Squared approximation may be incorrect due to low numbers; NA: no comparison could be made

The frequencies of A117A and 24 bp deletion were not statistically different from the control data.

Analysis of the genotype frequencies of D202D and E219K could not be done as they were only found in vCJD and not in the controls, as the latter were not sequenced.

## Discussion

Analysis of *PRNP *in sCJD, vCJD and controls has highlighted the clear primary role in disease susceptibility for the codon 129 genotype and has provided a base-line for the healthy population for future vCJD susceptibility predictions. In addition, the presence of genetic variation (D202D and E219K) in vCJD that is not seen in sCJD suggests possible additional risk factors. A novel non-synonymous polymorphism (D167G) in a case of suspected sCJD has also underlined the need for *PRNP *genetic screening of patients.

For accurate diagnosis of the various forms of human CJD, and especially to identify inherited genetic forms, DNA sequence analysis of the *PRNP *open reading frame (ORF) is necessary. Aside from the detection of any pathogenic mutations, this provides data on any polymorphic residues present, important for full characterisation of non-genetic cases [18]. The majority of non-pathogenic polymorphisms are rare.

**Codon 129 **genotype frequency data show the clear difference between CJD cases and controls with a reduction in the frequency of MV (21.4%) for sCJD cases compared to the controls (~45%), and 100% MM cases in vCJD. As the control genotype frequencies were not found to vary by age the case data is unlikely to be due to the significant difference in average age at onset between vCJD and sCJD. If clinical vCJD continues to manifest only in MM genotype individuals then the mathematical models for predicting the total epidemic size can use the population MM frequency of 44.1% in the UK population, generated by this study.

Other than M129V the two most common *PRNP *variants in Caucasian populations are: **A117A **(frequency ~5%), and a **24 bp deletion **in the repeat expansion region (~1%). Full *PRNP *sequencing of 118 confirmed UK vCJD patients found five with *PRNP *polymorphisms: two with E219K, two with D202D, and one with a 24 bp deletion (DelR34). The codon 117 polymorphism has not been found, as this is linked to the valine allele at codon 129, and all vCJD cases to date have been 129-MM.

**D202D **is a synonymous polymorphism and therefore there is no resulting change in prion protein amino acid sequence. No data are available on normal population frequency and the only record of its appearance has been in single French cases of sCJD and iCJD related to human Growth Hormone treatment (Dr N Delasnerie-Laupretre - personal communication). There are no structural changes to the prion protein itself and so this DNA sequence alteration would not directly affect protein folding kinetics in a potentially disease causing manner. Missense DNA sequence change GAC (glutamic acid) to AAC (asparagine) at this codon (D202N) is associated with a disease phenotype of Gerstmann-Sträussler-Scheinker syndrome (GSS) a genetic form of human prion disease [31]. The D202D change may be linked with a haplotype, as yet undefined, that alters susceptibility via an alternative route such as differential expression of PrP. This may possibly be the reason why ~2% of tested vCJD cases have been found with this polymorphism. Until control population frequencies are available this possibility remains hypothetical.

**E219K **is a non-synonymous, missense mutation with a change in amino acid and, therefore, the possibility of protein structural differences which may be linked to an increase in disease susceptibility. This polymorphism has not been found in Western European populations and has only been investigated in detail, with regards to CJD, in Japan [32] and Korea [33] where approximately 12% and 8% respectively of the population carry the lysine (K) allele. These studies indicated that E219K influenced the clinical phenotype of GSS in cases with the P102L mutation [34], and found it was a protective factor to sCJD as no confirmed cases of sCJD in Japan or Korea carry the polymorphism. In the two vCJD cases found in our study with E219K, there were no clinico-pathological differences from the other vCJD cases. These patients were of non-Caucasian origin, therefore their genetic ancestry may originate from populations where E219K is more common than the UK [29]. There remains a possibility that the presence of this polymorphism may increase susceptibility to vCJD. A transgenic mouse model suggests that the lysine (K) allele PrP^C ^is more susceptible than the glutamic acid (E) allele to conversion to PrP^Sc ^[35].

**24 bp deletions **were found in healthy individuals, sCJD, and vCJD patients at similar frequencies. It is therefore proposed that this genetic variation does not influence the disease state or susceptibility, even though the PrP^C ^formed is significantly different at the metal binding domain of the N-terminus, as shown by other studies [36,37]. Deletion of two 24 bp repeats in this region has been linked to a familial form of CJD [38].

There have been no reported instances of the **D167G **sCJD polymorphism associated with familial human prion disease. Evidence for this event to be the cause of a genetic form of CJD in this patient is twofold:

1. The replacement of a large hydrophilic amino acid for a small hydrophobic one may disturb the physical properties of the prion protein and thus accelerate formation of the disease associated form (PrP^Sc^).

2. Transgenic mouse studies propose that codon 167 may be in a region of the prion protein structure where chaperone molecules bind and accelerate disease transmission [39].

Without the additional evidence of further affected members of the family with the mutation or data on a healthy control population there is no direct proof of the association of D167G and CJD.

## Conclusions

This study has produced genotype frequencies for the most common *PRNP *genetic variants in the largest cohort of healthy individuals from the UK so far published, and in groups of patients with vCJD and sCJD. These data can act as a benchmark for studying the genotype frequency variation found in human prion disease in the UK. DNA sequence analysis of vCJD patients has revealed the extent of genetic variation within this population to include potential new risk factors, and sCJD analysis has uncovered a novel *PRNP *polymorphism.

## Competing interests

The authors declare that they have no competing interests.

## Authors' contributions

MTB, RGW, RSGK designed the study. MTB performed the genotyping. CP and CAH assessed the clinical presentation of vCJD and sCJD patients with *PRNP *polymorphisms. All authors helped draft and approve the final manuscript.

## Pre-publication history

The pre-publication history for this paper can be accessed here:

http://www.biomedcentral.com/1471-2350/10/146/prepub
